# Evaluating the Impact of Eating Disorder Early Intervention on Young People's Work, Education and Social Functioning

**DOI:** 10.1002/erv.70029

**Published:** 2025-08-29

**Authors:** Lucy Gallagher, Karina L. Allen, Ulrike Schmidt

**Affiliations:** ^1^ Centre for Research in Eating and Weight Disorders Department of Psychological Medicine Institute of Psychiatry Psychology and Neuroscience King's College London London UK; ^2^ South London and Maudsley NHS Foundation Trust London UK

**Keywords:** early intervention, eating disorder services, feeding and eating disorders, national health services, productivity

## Abstract

**Background:**

Eating disorders (EDs) impair education, work and social functioning. First Episode Rapid Early Intervention for Eating Disorders (FREED) is a nationally implemented early intervention service model in England for young people (16–25 years) with an ED duration of three years or less. This study evaluated the impact of FREED on functional outcomes using data from the multi‐site FREED‐Upscaled (FREED‐Up) study.

**Methods:**

A longitudinal design analysed data from 278 patients recruited in 2017–2018 across four specialist ED services. Work, education and social functioning were assessed using the Psychological Outcome Profile (PSYCHLOPS) and the Work and Social Adjustment Scale (WSAS) at baseline, 3, 6 and 12‐month. ED psychopathology and depression/anxiety were also measured. Linear mixed models examined WSAS and PSYCHLOPS changes, with correlations calculated between outcomes.

**Results:**

At baseline, 63% of patients reported work/education or social‐related issues on the PSYCHLOPS. WSAS and PSYCHLOPS scores significantly improved over time, with greater long‐term gains in treatment completers. WSAS reductions correlated with improvements in other clinical measures.

**Conclusions:**

Treatment within FREED is associated with significant improvements in work, education, and social functioning, alongside clinical gains. These findings underscore the value of early intervention in reducing functional impairments in young people with EDs.

## Highlights

- Sixty‐three percent of patients with recent onset eating disorders reported difficulties in work, education, or social functioning.
- Treatment within the First Episode Rapid Early Intervention for Eating Disorders (FREED) model—an early intervention service in England for young people (16–25 years) with an ED duration of three years or less—was associated with improvements in patient‐reported work/education and social functioning.
- Improvements in work/education and social functioning correlated with reductions in eating disorder symptomatology, anxiety, and depression.

## 1 Introduction and Aims

Eating disorders (EDs), such as anorexia nervosa (AN), bulimia nervosa (BN) and binge eating disorder (BED), are severe mental disorders characterised by abnormal eating or weight‐control behaviours. These disorders impair physical health and disrupt psychosocial functioning, leading to disruptions in daily activities, social interactions, and professional roles (Treasure et al. 2020; American Psychiatric Association 2013).

There is substantial evidence highlighting the impact of EDs on occupational outcomes. A community survey across 14 upper‐middle‐ and high‐income countries (*N* = 24,124) found that among individuals who met criteria for BN or BED in the past 12 months, 54.5% and 46.7%, respectively, reported role impairment. Severe role impairment was reported by 21.8% of those with 12‐month BN and 13.2% with 12‐month BED (Kessler et al. 2013). A longitudinal study found that 25% of young adults with AN (*N* = 51) were unemployed due to psychiatric disability, with all individuals experiencing persistent EDs remaining unemployed (Wentz et al. 2009). Furthermore, reliance on social welfare is common among patients with EDs (Treasure et al. 2015; Lindblad et al. 2006).

Globally, EDs impose significant economic burdens, partly due to reduced task completion and absenteeism. In the US, productivity losses accounted for $48.6 billion of the $64.7 billion in ED‐related costs (2018–2019), with AN incurring the highest individual losses ($21,496 per person) (Streatfeild et al. 2021). In the UK, 2017 estimates placed the annual cost of BEDs at three to five billion pounds, with 40% reporting work absences and 60% missing work or education days (Jenkins 2022).

Individuals with EDs also often experience social impairments. Studies indicate that even after recovery, many report smaller social networks and lower frequencies of social contact (Striegel‐moore et al. 2003). Interpersonal difficulties are integral to ED maintenance, as described in the Cognitive Interpersonal Maintenance Model (Treasure, Willmott, et al. 2020). Therapeutic approaches such as MANTRA (Maudsley Model of Anorexia Nervosa Treatment for Adults) explicitly aim to address these relational challenges (U. Schmidt et al. 2012).

In England, the First Episode Rapid Early Intervention for Eating Disorders (FREED) model is a leading early intervention approach (Mills et al. 2024). FREED provides timely access to evidence‐based treatment tailored to the early stages of the illness (defined as a duration of untreated ED [DUED] of three years or less) and the developmental needs of young people (emerging adults; ages 16–25). Detailed descriptions of the FREED service model and care pathway can be found elsewhere (Fukutomi et al. 2020). Early evidence supporting FREED was derived from the FREED‐Up (FREED‐Upscaled) study conducted in four specialist ED services (Austin et al. 2022; Flynn et al. 2021). The national rollout of FREED began in early 2020 and was completed by March 2023, with FREED now operational in 54 National Health Service (NHS) Trusts across England.

Previous analyses in the FREED‐Up sample have demonstrated improvements in work and social functioning as measured by the Work and Social Adjustment Scale (WSAS) (Austin et al. 2022). Additionally, the individualised patient‐reported outcome measure, PSYCHLOPS, has been validated in this sample, capturing concerns not addressed by traditional ED measures in 53.3% of participants (Austin et al. 2021). However, the specific domains that emerged from the PSYCHLOPS—particularly those related to work and social difficulties—have not yet been examined in detail. Furthermore, it remains unknown how these concerns evolve with treatment within FREED or how changes in PSYCHLOPS scores correspond to changes in WSAS scores. Investigating this relationship is crucial for evaluating the efficacy of treatment within FREED in patient‐centred terms, with the ultimate goal of supporting patients in returning to work or education.

The current study aimed to use FREED‐Up data to answer the following research questions:

*Research Question (RQ) 1*: What proportion of patients reported work, education, or social‐related issues as problems, as measured by the PSYCHLOPS?

*RQ2*: Does treatment in the FREED pathway lead to an improvement in patient‐reported work and social functioning, measured by the WSAS and PSYCHLOPS?

*RQ3*: How do changes in WSAS score relate to other clinical outcome measures (e.g., depression/anxiety symptoms) over time?

## 2 Method

### 2.1 Design and Sample

Ethical approval by all relevant committees was obtained (Austin et al. 2022). The study was conducted in accordance with the Declaration of Helsinki (World Medical Association 2013).

Full details regarding the FREED‐Up study can be found in Flynn et al. (2021). In brief, this study uses data from the FREED‐Upscaled (FREED‐Up) study, a longitudinal quasi‐experimental evaluation (Austin et al. 2022; Flynn et al. 2021). Of the 502 patients in the dataset, only those seen through the FREED pathway were included (*n* = 278). These patients were aged 16–25 with a primary DSM‐5 ED diagnosis and an illness duration of under three years. Participants were recruited between January 2017 and September 2018 from referrals to four specialist outpatient ED services in England: South London and Maudsley NHS Foundation Trust (SLaM), Central and North West London NHS Foundation Trust (CNWL), North East London NHS Foundation Trust (NELFT), and Leeds and York Partnership NHS Foundation Trust (LYPFT). FREED‐Up patients completed a baseline (T1) set of questionnaires, which included demographic questions and widely recognised outcome measures. These questionnaires were administered again at 3 (T2), 6 (T3), and 12 months (T4) following the baseline.

### 2.2 Outcomes

#### 2.2.1 Psychological Outcome Profiles (PSYCHLOPS)

For RQ1, we used the Psychological Outcome Profiles (PSYCHLOPS), an individualised outcome measure, designed to capture patient‐identified concerns most relevant to them. There are three versions of PSYCHLOPS (pre‐therapy [T1], during therapy [T2 and T3], and post‐therapy [T4]). At T1, participants were asked to identify two main problems that had been troubling them and one activity that was difficult to do. Each was rated on a six‐point Likert scale (0 = not at all affected; 5 = severely affected). Participants often distributed work‐, education‐, and social‐related concerns across the three problem‐focused items. As such, we treated all problem/difficulty responses as part of a unified ‘problem’ domain. At each follow‐up timepoint, participants re‐rated the same problems identified at T1. From T2 onwards, they were also invited to add a new problem, which could vary across timepoints. PSYCHLOPS has good internal consistency (*α* = 0.82) (Sales et al. 2023). The PSYCHLOPS questionnaire is available at: http://www.psychlops.org.uk/versions.

#### 2.2.2 The Work and Social Adjustment Scale (WSAS)

WSAS measures impairment in work/education and social functioning due to illness, in this context ED. WSAS scores can range from 0 to 40 and be classified as < 10 = subclinical impairment, 10–20 = significant functional impairment, and > 20 = moderately severe impairment. WSAS demonstrates strong internal consistency (*α* = 0.70–0.94) (Mundt et al. 2002).

#### 2.2.3 Eating Disorder Examination Questionnaire (EDE‐Q)

The Eating Disorder Examination Questionnaire (EDE‐Q; Fairburn and Beglin 2008) is a self‐report measure of ED psychopathology. Patients rate the frequency or severity of symptoms over the past 28 days on a seven‐point Likert scale, with higher scores (out of 6) indicating greater impairment. EDE‐Q has good internal consistency (*α* = 0.70–0.93) (Jennings and Phillips 2017).

#### 2.2.4 Clinical Outcomes in Routine Evaluation‐10/Outcome Measure (CORE‐10/OM)

The Clinical Outcomes in Routine Evaluation‐10/Outcome Measure (CORE‐10/OM; Barkham et al. 2013) is a 10‐item measure of general psychological distress, with strong internal consistency (*α* = 0.90). Each item is rated on a 5‐point scale, with higher total scores (out of 40) indicating greater symptom severity.

#### 2.2.5 BMI

Body mass index (BMI; kg/m^2^) was measured at each timepoint via questionnaire.

### 2.3 Analyses

For RQ1, PSYCHLOPS statements were categorised according to the coding criteria detailed in Supporting Information S1: Table 1. Data were extracted for patients who rated work, education, or social issues as a problem on the PSYCHLOPS at T1. Work and Education categories were combined into a single Work/Education category as education often represents the primary form of work for younger populations. The number and proportion of patients who reported work/education, or social‐related issues, or both, as a problem at T1 were calculated. The number and proportion of patients who reported these issues as an additional problem at T2, T3, and T4 was also calculated.

To assess if treatment in the FREED pathway leads to an improvement in WSAS and PSYCHLOPS scores, linear mixed models (LMMs) were used. Logistic regression examined predictors of missingness (diagnosis, age at assessment, treatment completion, gender, ethnicity, BMI at assessment, T1/T2 WSAS and EDE‐Q score). Treatment completion was the only significant predictor of missingness and was therefore included as a covariate in the models. Time was modelled as a fixed effect. For the WSAS model, Service (to account for clustering within treatment sites) and Patient ID (to account for repeated measures) were included as random effects. For the PSYCHLOPS model, ANOVA model comparisons indicated that including Service as a random effect did not improve model fit, as its variance estimates approached zero. Therefore, only Patient ID was retained as a random effect. This model included only patients who had identified work/education, or social‐related issues as a concern at T1.

For RQ3, correlation matrices were calculated between all measures at each time point to examine how changes in WSAS scores relate to other clinical outcomes.

## 3 Results

### 3.1 Sample

Table 1 presents the demographic and clinical characteristics of the patients included in the study at each time point. A participant flow diagram can be found in Supporting Information S1: Appendix 1.

**TABLE 1:** Characteristics of FREED‐Up patients at each time point.

	T1	T2	T3	T4
Mean age (years) (SD)	20.19 (2.39)			
Mean DUED (months) (SD)	17.85 (10.38)			
Mean WSAS (SD)	20.68 (8.79)	17.27 (9.48)	14.57 (10.03)	12.30 (10.45)
Mean BMI (SD)	19.93 (4.40)	19.72 (4.37)	20.20 (4.95)	21.05 (4.70)
Mean EDE‐Q (SD)	4.08 (1.21)	3.17 (1.47)	2.85 (1.57)	2.31 (1.55)
Mean CORE‐10/OM (SD)	19.66 (7.53)	16.86 (8.37)	14.29 (7.58)	13.26 (8.81)
Mean PSYCHLOPS score (SD) (for those citing Work/Education/Social Issues at T1 only, *n* = 175)	4.09 (1.06)	2.96 (1.51)	2.51 (1.57)	2.02 (1.83)
Diagnosis % (*n*)				
Anorexia nervosa	42.08 (117)			
Bulimia nervosa	25.90 (72)			
Binge eating disorder	1.08 (3)			
Other specified feeding or eating disorder	30.94 (86)			
Data missingness % (*n*)				
DUED	3.96 (11)			
BMI	0.72 (2)	20.86 (58)	29.86 (83)	39.21 (109)
EDE‐Q	0 (0)	22.30 (62)	34.53 (96)	37.05 (103)
CORE‐10/OM	0.36 (1)	22.30 (62)	34.53 (96)	37.05 (103)
WSAS	0 (0)	22.30 (62)	34.53 (96)	37.05 (103)
PSYCHLOPS score (for those citing work/education/social issues at T1 only, *n* = 175)	0 (0)	25.84 (46)	41.71 (73)	41.14 (72)

Abbreviations: BMI, body mass index (BMI; kg/m^2^); CORE‐10/OM, clinical outcomes in routine evaluation‐10/outcome measure; EDE‐Q, eating disorder examination questionnaire; DUED, duration of an untreated eating disorder, FREED; first episode rapid early intervention for eating disorders, PSYCHLOPS; psychological outcome profiles; WSAS, work and social adjustment score.

### 3.2 How Many Patients Reported Work/Education and Social‐Related Issues at Each Time Point?

62.95% (175) patients identified a work/education issue, a socio‐emotional/interpersonal issue, or both as a problem at T1. From T2 onwards, patients were able to add one additional problem to the original three they had described at T1. The number of patients reporting these concerns at each time point are summarised in Table 2. For example, one patient described:

I can't work full‐time as I get tired easily and feel sick often. The lunch breaks are too far away, and I feel sick when I have been hungry for a while.

**TABLE 2:** The number and percentage of patients reporting work/education and/or social‐related issues as problems at each time point.

Time point	Category	Count (% of total sample)
T1	Work/education	41 (14.75)
	Socio‐emotional/interpersonal	116 (41.73)
	Both work/education & socio‐emotional	18 (6.47)
T2	Work/education	11 (3.96)
	Socio‐emotional/interpersonal	6 (2.16)
T3	Work/education	6 (2.16)
	Socio‐emotional/interpersonal	5 (1.80)
T4	Work/education	5 (1.80)
	Socio‐emotional/interpersonal	7 (2.52)

Another patient expressed challenges with academic performance:

Concentration and motivation for exams.

Similarly, socio‐emotional and interpersonal concerns were prevalent, with patients highlighting difficulties in maintaining social relationships, particularly in contexts involving food:

Socialising, particularly when plans are made last minute and revolve around food and drink.

### 3.3 Does Treatment in the FREED Pathway Lead to an Improvement in Work and Social Functioning, Measured by WSAS and PSYCHLOPS?

At T1, 50.72% of patients were classified as experiencing moderately severe clinical impairment on the WSAS. By T4, 48% of patients fell into the sub‐clinical impairment category (Table 3).

**TABLE 3:** WSAS scores by clinical category for FREED‐Up patients over the 4 time points.

Time point	Sub‐clinical impairment % (*n*)	Significant impairment, % (*n*)	Moderately severe impairment, % (*n*)
T1	10.07 (28)	39.21 (109)	50.72 (141)
T2	25.93 (56)	29.63 (64)	44.44 (96)
T3	37.91 (69)	26.92 (49)	35.16 (64)
T4	48.00 (84)	26.86 (47)	25.14 (44)

**FIGURE 1:**
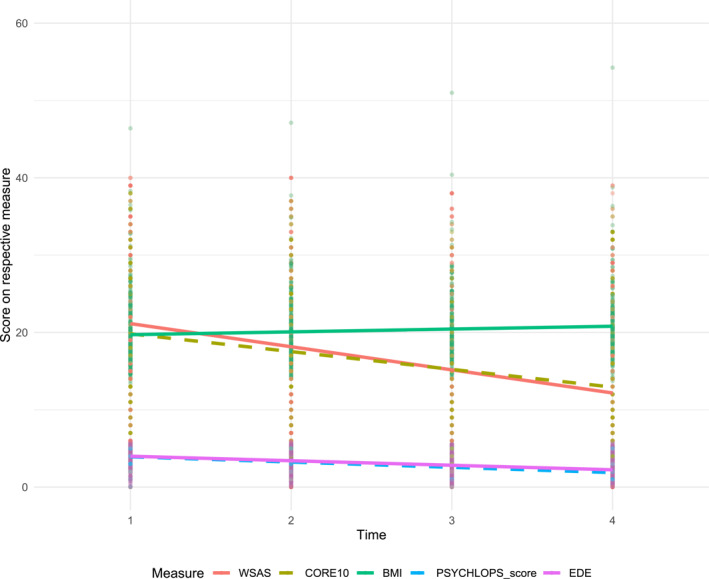
WSAS, PSYCHLOPS, CORE‐10, EDE‐Q Scores and BMI for FREED‐Up patients over the 4 time points. These outcome measures are plotted on the same vertical axis, but they operate on different scales: BMI (Body Mass Index; kg/m^2^): Typically ranges from 10 to 40+ in clinical populations; CORE‐10 (Clinical Outcomes in Routine Evaluation—10): Scores range from 0 to 40; EDE‐Q (Eating Disorder Examination Questionnaire): Scores range from 0 to 6; WSAS (Work and Social Adjustment Scale): Scores range from 0 to 40; PSYCHLOPS: Scores range from 0 to 5.

The LMM for WSAS scores showed a significant improvement in functional impairment over time (Supporting Information S1: Figure 1). Compared to T1, WSAS scores decreased significantly at all subsequent time points: T2 (*β* = −7.45, SE = 1.15, t (824.49) = −6.47, *p* < 0.001), T3 (*β* = −8.53, SE = 1.38, t (826.10) = −6.17, *p* < 0.001), and T4 (*β* = −5.66, SE = 1.33, t (832.24) = −4.26, *p* < 0.001). Although treatment completion was not a significant main effect, it interacted with time. Individuals who completed treatment initially had higher WSAS scores than non‐completers at T2 (*β* = 5.33, SE = 1.31, t (809.10) = 4.08, *p* < 0.001) and T3 (*β* = 3.28, SE = 1.53, t (812.76) = 2.15, *p* = 0.032). However, completers demonstrated greater long‐term improvements, as evidenced by a significant decline in WSAS scores by T4 (*β* = −3.67, SE = 1.49, t (816.89) = −2.47, *p* = 0.014).

A second LMM indicated significant reductions in PSYCHLOPS scores over time. Compared to T1, PSYCHLOPS scores were significantly lower at T2 (*β* = −0.99, SE = 0.24, t (614.41) = −4.09, *p* < 0.001), T3 (*β* = −2.10, SE = 0.29, t (625.83) = −7.37, *p* < 0.001), and T4 (*β* = ‐ SE = 0.28, t (630.66) = −2.65, *p* < 0.001). Again, the main effect of treatment completion was not significant, but an interaction with time was observed. At T2 and T3, there were no significant differences in PSYCHLOPS scores between treatment completers and non‐completers. However, by T4, treatment completers had significantly lower PSYCHLOPS scores than non‐completers (*β* = −1.71, SE = 0.31, t (617.03) = −5.46, *p* < 0.001), suggesting greater improvement over time. Model robustness was confirmed by bootstrapped confidence intervals (1000 resamples), which closely matched the Wald estimates (Supporting Information S1: Table 2).

WSAS and PSYCHLOPS assess overlapping but distinct aspects of functional impairment. As expected, they were moderately correlated at T1 (*r* = 0.52, 95% CI: 0.42–0.61, *p* < 0.001), indicating some shared variance. Given their conceptual distinction, separate models were used for each outcome. Although this increases the potential for inflated Type I error, the consistency of findings across both measures supports the robustness of results and suggests they reflect meaningful patterns rather than chance.

### 3.4 How Do Changes in WSAS Score Relate to Other Clinical Outcome Measures Over Time?

Figure 1 illustrates WSAS scores over time in comparison to other clinical outcome measures: PSYCHLOPS, CORE‐10, EDE‐Q, and BMI. Descriptively, these measures covary, with reductions in WSAS, PSYCHLOPS, CORE‐10, and EDE‐Q scores, and concurrent increases in BMI, indicating improvements across domains. These patterns reflect associations rather than directional effects. Specific correlations at each time point are provided in Supporting Information S1: Table 3.

## 4 Discussion

This study evaluated the impact of treatment via FREED on work, education, and social difficulties among emerging adults with recent‐onset EDs. At baseline, 62.95% of patients reported difficulties in these domains (14.75% work/education‐related, 41.73% social, 6.47% both), reflected in high baseline WSAS scores. Both PSYCHLOPS and WSAS scores improved significantly over time—particularly among treatment completers. These improvements were observed alongside reductions in ED and general psychopathology.

Our results align with previous research demonstrating that effective ED treatments often lead to improvements in work and social functioning. For example, significant WSAS score reductions were observed following inpatient and day programs (Li et al. 2020). Similarly, functional improvement has been observed in less intensive interventions such as guided self‐help cognitive behavioural therapy (CBT) for BED (Striegel‐Moore et al. 2010). In the context of early intervention, previous evidence has shown general improvements in PSYCHLOPS and WSAS following FREED treatment (Austin et al. 2022), however, the present study is the first to specifically assess individualised patient‐reported outcomes related to work, education, and social issues on the PSYCHLOPS.

Treatment completers demonstrated greater improvements in both WSAS and PSYCHLOPS scores. Given that dropout is a well‐documented challenge in ED treatment (e.g., Schnicker et al. 2013), targeted strategies are needed to reduce attrition and optimise outcomes. Identifying patients at higher risk of premature dropout—such as those with greater psychiatric comorbidity (Vall and Wade 2015)– could allow clinicians to tailor treatment approaches based on individual dropout risk and motivation for recovery. Implementing such personalised approaches in ED treatment may help improve retention and maximise the benefits of early intervention programs like FREED (U. H. Schmidt et al. 2025).

A key strength of this study is its mixed‐methods approach to measuring patient‐reported work and social functioning. Both the quantitative WSAS scale and the qualitative PSYCHLOPS measure were used, allowing for a more comprehensive evaluation of FREED's impact in patient‐centred terms. However, a limitation is that both WSAS and PSYCHLOPS rely on self‐reported data, which may be subject to biases such as social desirability. In clinical practice, self‐report measures are often the most practical option, but future research could extend these findings by leveraging data linkage to assess the impact of FREED on employment outcomes, such as pay and employment status—an approach recently used in NHS Talking Therapies for Anxiety and Depression (TTad) (Office for National Statistics (ONS) 2024). Links with educational status and achievement could also be considered. Furthermore, self‐reported height and weight were used to calculate BMI. Although this approach facilitated consistent data collection across multiple time points, including remote follow‐up, self‐reported data can be subject to systematic error.

Another limitation is that the WSAS and PSYCHLOPS were not adapted for younger participants, which may have made some items less clear. Future studies may benefit from age‐specific adaptations when using these measures with adolescent populations.

Finally, these analyses were not pre‐registered. While our approach was informed by prior literature, the absence of a pre‐registered protocol introduces a risk of potential bias in interpretation.

## 5 Conclusions

Following the COVID‐19 pandemic, the UK has faced a prolonged slowdown in productivity growth, with more persistent challenges than other advanced economies (Lee and Parry 2025). While many factors contribute to this trend, the role of mental health cannot be overlooked, with previous research highlighting the substantial productivity losses linked to EDs (Streatfeild et al. 2021; Jenkins 2022). This study highlights the potential of FREED to improve work and social functioning by supporting earlier recovery and reducing life disruption. These findings point to the broader potential economic and social gains of early effective ED treatment.

## Author Contributions

**Lucy Gallagher:** conceptualization, data curation, formal analysis, investigation, methodology, visualization, writing – original draft, writing – review and editing. **Karina L. Allen:** conceptualization, methodology, project administration, supervision, writing – review and editing. **Ulrike Schmidt:** conceptualization, methodology, project administration, supervision, writing – review and editing.

## Ethics Statement

Data were collected under a data‐sharing agreement between South London and Maudsley NHS Foundation Trust and participating FREED services. Patients were informed of their right to opt out of data sharing via a privacy notice and information sheet. All data were de‐identified by participating services before sharing, ensuring that no personally identifiable information was included.

## Conflicts of Interest

The authors declare no conflicts of interest.

## Supporting information


Supporting Information S1

## Data Availability

The data used in this study is not publicly available.
