# Supplementary material for: Evaluating the Impact of Eating Disorder Early Intervention on Young People's Work, Education and Social Functioning
Source: Eur Eat Disord Rev. 2025 Aug 29;34(1):288–95. doi: 10.1002/erv.70029 (PMC12694690; doi:10.1002/erv.70029)
Supplement: Supplementary file 1 — Supporting Information S1 [file ERV-34-288-s001.docx]

**Appendices to Evaluating the Impact of Eating Disorder Early Intervention on Young People’s Work, Education, and Social Functioning**

**Appendix 1:** *Participant flow diagram*

**Supplementary Table 1:** *Coding Criteria for Work, Education, and Social Functioning Issues Reported by Patients in PSYCHLOPS Measure*.

**Supplementary Figure 1:** *Predicted WSAS Scores by Site****.***

**Supplementary Table 2:** *Fixed Effects Estimates and Confidence Intervals (CI) for PSYCHLOPS Scores*

**Supplementary Table 3:** *Pearson correlation coefficients between WSAS, PSYCHLOPS, CORE-10, EDE-Q Scores and BMI for FREED-Up patients over the 4 time points.*

**Appendix 1**

*Participant flow diagram*

*
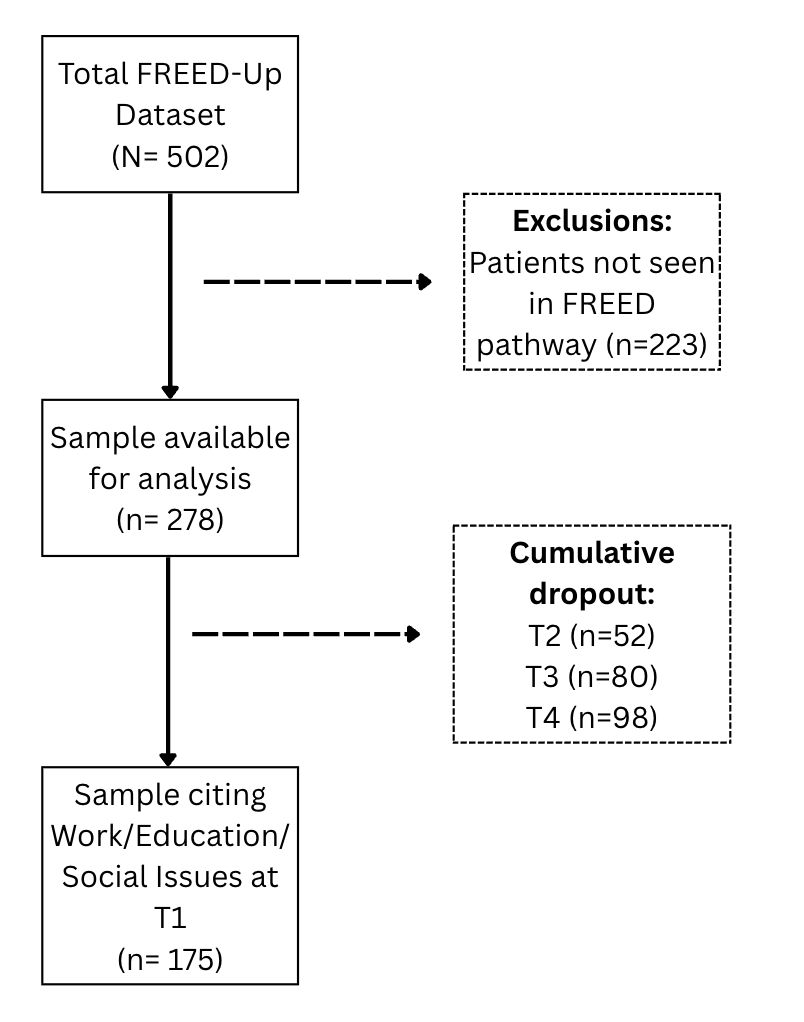
*

**Supplementary Table 1**

*Coding Criteria for Work, Education, and Social Functioning Issues Reported by Patients in PSYCHLOPS Measure*.

| **Content** | **Definition** | **Example** |
| --- | --- | --- |
| Work issues | Patient-generated outcomes referring to difficulties in attending or succeeding at work, or fears of job loss.  Includes concerns related to eating at work.  Excludes outcomes mentioning general lack of motivation or focus without specific reference to work.  Excludes outcomes related to personal passion projects or housework. | “I feel like I'm bad at my job” |
| Education issues | Patient-generated outcomes referring to difficulties in attending or succeeding at school, college, or university.  Includes concerns about lack of support during the transition to university.  Includes concerns related to eating at school or university.  Excludes outcomes mentioning general lack of motivation without specific reference to school. | “Lack of attendance in university as a result of the ED”. |
| Socio-emotional / interpersonal issues | Patient-generated outcomes referring to difficulties in attending or enjoying social activities, including eating with others.  Includes difficulties in generating or maintaining close relationships or feeling like a burden to others. | “That I don’t have any friends” |

**Supplementary Figure 1**

*Predicted WSAS Scores by Site****.***


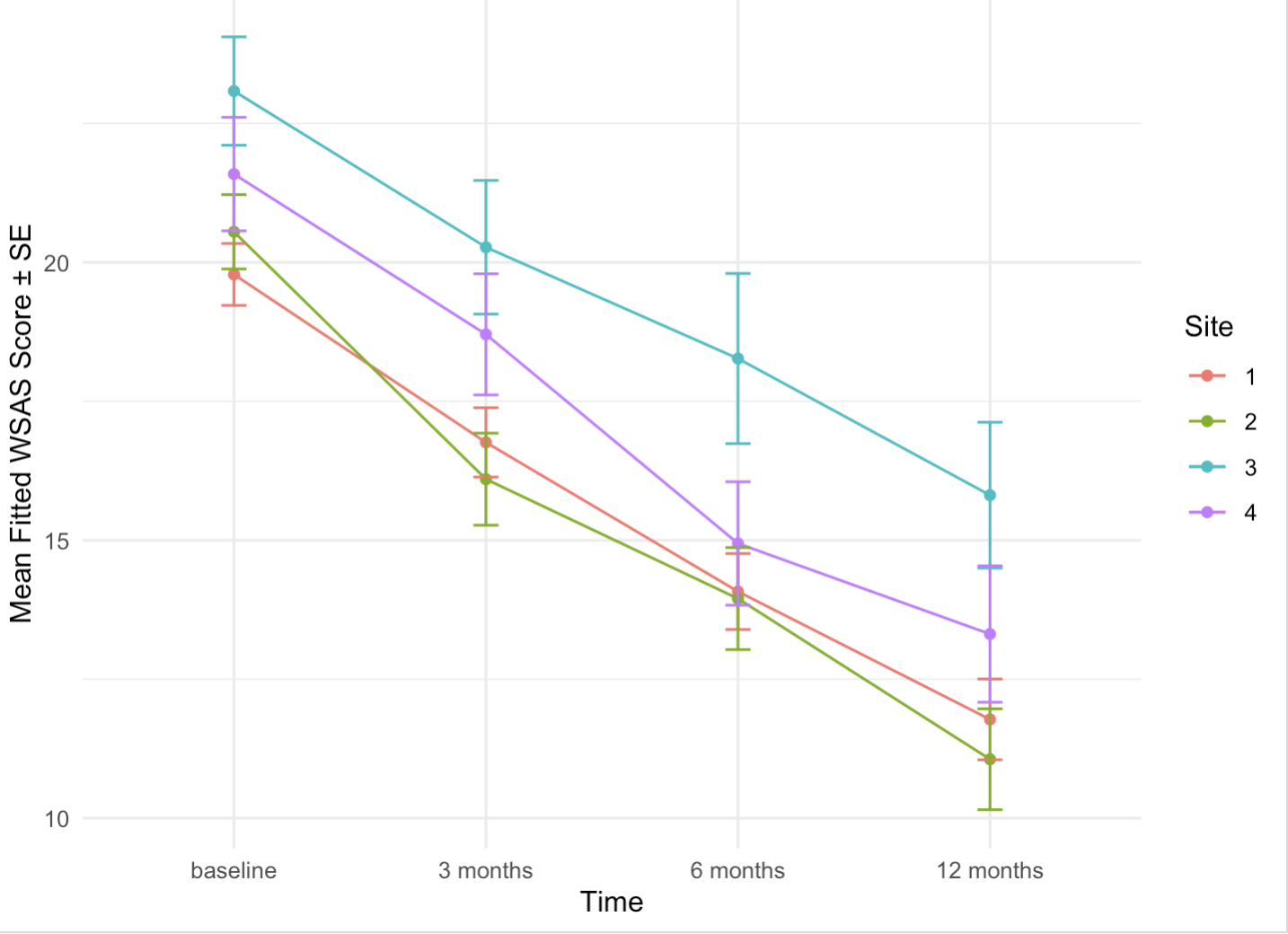


**Supplementary Table 2:**

*Fixed Effects Estimates and Confidence Intervals (CI) for PSYCHLOPS Scores*

| **Predictor** | **β (Estimate)** | **95% Wald CI** | **95% Bootstrapped CI** |
| --- | --- | --- | --- |
| (Intercept) | 3.89 | [3.52, 4.26] | [3.53, 4.28] |
| T2 | -0.99 | [-1.47, -0.52] | [-1.50, -0.49] |
| T3 | -2.10 | [-2.66, -1.54] | [-2.65, -1.57] |
| T4 | -0.74 | [-1.29, -0.19] | [-1.30, -0.21] |
| Treatment_complete | 0.28 | [-0.16, 0.78] | [-0.18, 0.74] |
| Treatment_complete x T2 | -0.25 | [-0.80, 0.30] | [-0.82, 0.32] |
| Treatment_complete x T3 | 0.56 | [-0.07, 1.18] | [-0.06, 1.22] |
| Treatment_complete x T4 | -1.71 | [-2.32, -1.09] | [-2.30, -1.13] |

**Supplementary Table 3:**

*Pearson correlation coefficients between WSAS, PSYCHLOPS, CORE-10, EDE-Q Scores and BMI for FREED-Up patients over the 4 time points.*

|  | **WSAS** | **EDE** | **CORE-10** | **BMI** | **PSYCHLOPS** |
| --- | --- | --- | --- | --- | --- |
| **T1** | | | | | |
| **WSAS** | 1.00 | 0.41***** | 0.56***** | -0.02 | 0.52***** |
| **EDE** | 0.41***** | 1.00 | 0.46***** | 0.19***** | 0.39***** |
| **CORE-10** | 0.56***** | 0.46***** | 1.00 | 0.07 | 0.37***** |
| **BMI** | -0.017 | 0.19***** | 0.07 | 1.00 | 0.05 |
| **PSYCHLOPS** | 0.52***** | 0.39***** | 0.37***** | 0.05 | 1.00 |
| **T2** | | | | | |
| **WSAS** | 1.00 | 0.65***** | 0.59***** | -0.03 | 0.44***** |
| **EDE** | 0.65***** | 1.00 | 0.55***** | 0.06 | 0.46***** |
| **CORE-10** | 0.59***** | 0.55***** | 1.00 | -0.06 | 0.47***** |
| **BMI** | -0.03 | 0.06 | -0.06 | 1.00 | 0.02 |
| **PSYCHLOPS** | 0.44***** | 0.46***** | 0.47***** | 0.02 | 1.00 |
| **T3** | | | | | |
| **WSAS** | 1.00 | 0.66***** | 0.69***** | -0.03 | 0.63***** |
| **EDE** | 0.66***** | 1.00 | 0.64***** | 0.02 | 0.51***** |
| **CORE-10** | 0.67***** | 0.64***** | 1.00 | 0.02 | 0.59***** |
| **BMI** | -0.03 | 0.02 | 0.02 | 1.00 | -0.13***** |
| **PSYCHLOPS** | 0.63***** | 0.51***** | 0.59***** | -0.13 | 1.00 |
| **T4** | | | | | |
| **WSAS** | 1.00 | 0.74***** | 0.72***** | -0.12 | 0.64***** |
| **EDE** | 0.74***** | 1.00 | 0.71***** | 0.02 | 0.59***** |
| **CORE-10** | 0.72***** | 0.71***** | 1.00 | 0.00 | 0.71***** |
| **BMI** | -0.12 | 0.02 | 0.00 | 1.00 | -0.06 |
| **PSYCHLOPS** | 0.64***** | 0.59***** | 0.71***** | -0.06 | 1.00 |

*Notes: Correlations are interpreted as weak (|r| = .10–.29), moderate (|r| = .30–.49), and strong (|r| ≥ .50) (Cohen, 1988). p < .05 is indicated by an asterisk (*).*

*Abbreviations: BMI, Body Mass Index (BMI; kg/m2); CORE‐10/OM, Clinical Outcomes in Routine Evaluation‐10/Outcome Measure; EDE‐Q, Eating Disorder Examination Questionnaire, PSYCHLOPS; Psychological Outcome Profiles, Work and social adjustment score; WSAS.*
